# Vpu-Mediated Counteraction of Tetherin Is a Major Determinant of HIV-1 Interferon Resistance

**DOI:** 10.1128/mBio.00934-16

**Published:** 2016-08-16

**Authors:** Dorota Kmiec, Shilpa S. Iyer, Christina M. Stürzel, Daniel Sauter, Beatrice H. Hahn, Frank Kirchhoff

**Affiliations:** a Institute of Molecular Virology, Ulm University Medical Center, Ulm, Germany; b Department of Medicine, Perelman School of Medicine, University of Pennsylvania, Philadelphia, Pennsylvania, USA; c Department of Microbiology, Perelman School of Medicine, University of Pennsylvania, Philadelphia, Pennsylvania, USA

## Abstract

Human immunodeficiency virus type 1 (HIV-1) groups M, N, O, and P are the result of independent zoonotic transmissions of simian immunodeficiency viruses (SIVs) infecting great apes in Africa. Among these, only Vpu proteins of pandemic HIV-1 group M strains evolved potent activity against the restriction factor tetherin, which inhibits virus release from infected cells. Thus, effective Vpu-mediated tetherin antagonism may have been a prerequisite for the global spread of HIV-1. To determine whether this particular function enhances primary HIV-1 replication and interferon resistance, we introduced mutations into the *vpu* genes of HIV-1 group M and N strains to specifically disrupt their ability to antagonize tetherin, but not other Vpu functions, such as degradation of CD4, down-modulation of CD1d and NTB-A, and suppression of NF-κB activity. Lack of particular human-specific adaptations reduced the ability of HIV-1 group M Vpu proteins to enhance virus production and release from primary CD4^+^ T cells at high levels of type I interferon (IFN) from about 5-fold to 2-fold. Interestingly, transmitted founder HIV-1 strains exhibited higher virion release capacity than chronic control HIV-1 strains irrespective of Vpu function, and group M viruses produced higher levels of cell-free virions than an N group HIV-1 strain. Thus, efficient virus release from infected cells seems to play an important role in the spread of HIV-1 in the human population and requires a fully functional Vpu protein that counteracts human tetherin.

## IMPORTANCE

Understanding which human-specific adaptations allowed HIV-1 to cause the AIDS pandemic is of great importance. One feature that distinguishes pandemic HIV-1 group M strains from nonpandemic or rare group O, N, and P viruses is the acquisition of mutations in the accessory Vpu protein that confer potent activity against human tetherin. Adaptation was required because human tetherin has a deletion that renders it resistant to the Nef protein used by the SIV precursor of HIV-1 to antagonize this antiviral factor. It has been suggested that these adaptations in Vpu were critical for the effective spread of HIV-1 M strains, but direct evidence has been lacking. Here, we show that these changes in Vpu significantly enhance virus replication and release in human CD4^+^ T cells, particularly in the presence of IFN, thus supporting an important role in the spread of pandemic HIV-1.

## INTRODUCTION

Pandemic human immunodeficiency virus type 1 (HIV-1) emerged following the transmission of SIVcpz, a simian immunodeficiency virus (SIV) from chimpanzees (cpz), to humans early in the 20th century (1). Since then, this major (M) group of HIV-1 has infected more than 70 million people and caused more than 30 million deaths. In contrast, HIV-1 groups O, N, and P, which also resulted from zoonotic transmissions of chimpanzee (N) and gorilla (O and P) SIVs, have spread far less efficiently in the human population. Group O viruses have been found in about 100,000 individuals in Cameroon and surrounding countries (2), while HIV-1 group N and P viruses are rare and have been detected in only a few individuals (3, 4).

One possible reason for why only HIV-1 group M became pandemic is the acquisition of potent antitetherin activity by its Vpu protein (5). Tetherin is an antiviral restriction factor that inhibits virus release by tethering nascent virus particles to the surfaces of infected cells (6, 7). Most primate lentiviruses, including SIVcpz and SIVgor (gorilla), use their Nef protein to antagonize this antiviral factor (5, 8, 9). A deletion in the cytoplasmic domain of human tetherin, however, confers resistance to SIV Nef proteins (Nefs) and thus represents a significant barrier for successful zoonotic transmission (10, 11). Pandemic group M and (to a much lesser extent) rare group N strains acquired Vpu-mediated antitetherin activity (5, 12), while HIV-1 group O strains evolved the ability to counteract human tetherin by adapting their Nef protein to target a region adjacent to the deletion (13). However, neither of the two known group P viruses acquired significant anti-human tetherin activity (14, 15).

It has been shown that specific amino acid residues in the transmembrane domain (TMD) allow HIV-1 group M Vpu proteins (Vpus) to interact directly with the TMD of tetherin and to counteract this restriction factor (16–18). In contrast, other Vpu functions are conserved between HIV-1 and its simian precursors. For example, SIVcpz and SIVgor Vpus are active in degrading human CD4 (5) and in suppressing the transcription factor NF-κB and interferon induction in human cells (19). Furthermore, SIVcpz Vpu proteins downregulate cell surface expression of human natural killer, T, and B cell antigen (NTB-A) and CD1d (12), which suppress NK cell-mediated lysis of virally infected cells (20) and antigen presentation by virally infected dendritic cells, respectively (21). Complete abrogation of Vpu impairs HIV-1 replication in primary CD4^+^ T cells and humanized mice and renders the virus hypersensitive to alpha interferon (IFN-α) inhibition (22–24). However, it remains unknown how much the more recently acquired Vpu-mediated antitetherin activity contributes to replication fitness and IFN resistance of HIV-1 group M. To address this, we introduced mutations in the TMDs of the Vpu proteins of six group M infectious molecular clones (IMCs) that specifically abrogated their ability to antagonize human tetherin. We show that these changes significantly decrease HIV-1 replication and increase IFN sensitivity in primary human CD4^+^ T cells. Thus, human-specific adaptation of SIVcpz Vpu was likely required to gain maximal replication fitness of group M viruses in the new host and facilitate the successful colonization of humans.

## RESULTS

### Generation of HIV-1 Vpu mutants that are selectively impaired in tetherin antagonism.

**FIG 1:**
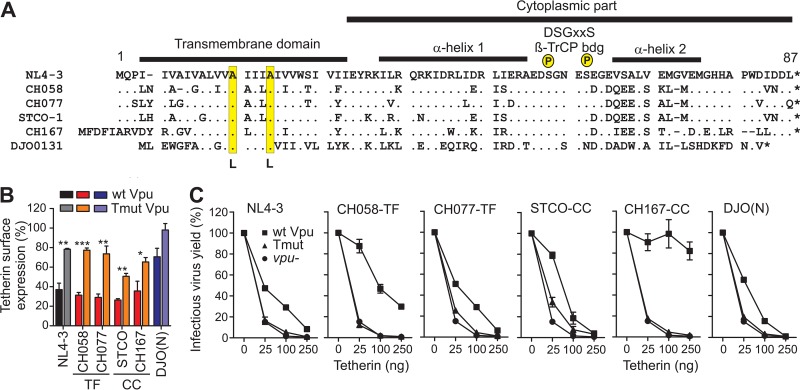
Mutant Vpus selectively defective in tetherin antagonism. (A) Alignment of Vpu amino acid sequences analyzed. The NL4-3 Vpu sequence is shown in the top row for comparison. Important functional domains are indicated above the sequences, and the mutated Ala residues are highlighted in yellow. Dots specify amino acid identity, and dashes represent gaps introduced to optimize the alignment. bdg, binding; P, phosphate. (B) Down-modulation of human tetherin by wild-type (wt) and mutant Vpu proteins in HEK293T cells cotransfected with vectors coexpressing eGFP and Vpu and a construct expressing human tetherin. Shown are the levels of tetherin cell surface expression relative to those measured in cells transfected with the control vector containing only eGFP (100%). Values are mean values (plus standard errors of the means [SEM] [error bars]) derived from three experiments. Wild-type Vpu alleles are color coded in dark colors, and mutant Vpus are shown in light colors. Values that are significantly different are indicated by asterisks as follows: *, *P* < 0.05; **, *P* < 0.01; ***, *P* < 0.001. (C) Virus release from HEK293T cells following transfection with *vpu*-defective HIV-1 NL4-3, expression constructs for the indicated Vpu proteins or eGFP only, and various amounts of plasmid expressing human tetherin. Infectious virus was determined by infection of TZM-bl indicator cells and is shown as a percentage of that detected in the absence of tetherin (100%). Infections were performed in triplicate, and the results were confirmed in an independent experiment.

Efficient counteraction of human tetherin by Vpu distinguishes HIV-1 group M strains from other group O, N, and P strains (5). To examine the effects of Vpu-mediated tetherin antagonism on HIV-1 replication and IFN sensitivity in human CD4^+^ T cells, we generated a panel of infectious molecular clones (IMCs) that lacked this specific Vpu function. We achieved this by mutating two alanines in the TMD of Vpu, which have previously been shown to be critical for antagonism of human tetherin (16–18), to leucines (Fig. 1A). These mutations were introduced into two transmitted founder (TF) (CH058-TF and CH077-TF) and two chronic control (CC) viruses (STCO-CC and CH167-CC) (25). The T-cell-line-adapted (CXC chemokine receptor 4 [CXCR4]-tropic) NL4-3 clone served as a control. All IMCs represented clade B viruses, except for CH167-CC, which is a clade C strain (Table 1). For comparison, we also generated a mutant of the group N HIV-1 clone DJO0131 (26) to determine whether the modest gain of antitetherin activity by this viral lineage (5, 12) is sufficient to promote virus replication and release in primary CD4^+^ T cells.

**TABLE 1:** Infectious molecular clones of HIV-1 analyzed^a^

HIV-1 clone	Group	Subtype	Type	Tropism	Vpu length (aa)	Mutations	Reference
NL4-3	M	B	LA	X4	81	A14L A18L	44
CH058	M	B	TF	R5	80	A14L A18L	32
CH077	M	B	TF	R5/X4	81	A15L A19L	32
STCO	M	B	CC	R5	81	A15L A19L	32
CH167	M	C	CC	R5	84	A20L A24L	32
DJO0131	N		nk	nk	74	A12L A16L	12

a Abbreviations: LA, laboratory adapted; TF, transmitted founder; CC, chronic control; nk, not known; aa, amino acids.

To verify that the introduced mutations abrogated Vpu’s ability to counteract human tetherin, we cotransfected HEK293T cells with vectors expressing wild-type (wt) or TMD mutant (Tmut) Vpu proteins and enhanced green fluorescent protein (eGFP) (or eGFP alone for control) together with a construct expressing human tetherin. The TMD mutations did not affect Vpu expression levels (see Fig. S1A in the supplemental material), but they significantly impaired the ability of all HIV-1 M Vpus to reduce tetherin cell surface expression (Fig. 1B and Fig. S1B). In agreement with published data (12), the DJO0131 group N Vpu was poorly expressed (Fig. S1A) and had only a modest effect on tetherin, which was entirely abolished by the TMD mutations (Fig. 1B and Fig. S1B). To further examine the effects of these TMD mutations, we analyzed the efficiency of virus release from HEK293T cells cotransfected with *vpu*-defective HIV-1 NL4-3 together with constructs expressing wt or TMD-mutated Vpu proteins or eGFP only, as well as plasmids expressing human tetherin at different doses. The results showed that the TMD mutations completely disrupted the ability of Vpu to enhance virus release (Fig. 1C). Notably, HIV-1 M subtype C CH167-CC Vpu antagonized tetherin more efficiently than subtype B and group N Vpus (Fig. 1C).

To determine the specificity of the TMD mutations for the antitetherin activity of Vpu, we transfected HEK293T cells with vectors coexpressing Vpu and eGFP together with constructs expressing CD4, NTB-A, or CD1d. All HIV-1 M Vpus strongly reduced cell surface expression of CD4, while the group N Vpu had little effect (see Fig. S1B and S1C in the supplemental material). Although only Vpus from the two CC HIV-1 M strains STCO-CC and CH167-CC significantly reduced NTB-A and CD1d cell surface expression (Fig. S1D and S1E), the effect of Vpu on these receptors was not significantly impaired by the TMD mutations. HIV-1 M Vpus also suppressed antiviral gene expression and immune activation by inhibiting NF-κB activation (19, 27). Cotransfection of HEK293T cells with vectors coexpressing Vpu and eGFP together with an NF-κB-dependent firefly luciferase reporter construct and a constitutively active mutant of IκB kinase β (IKKβ) showed that the CH058-TF, CH077-TF, and STCO-CC Vpus suppressed IKKβ-mediated NF-κB activation by ~80%, whereas the NL4-3 Vpu achieved ~40% inhibition and the HIV-1 N Vpu was inactive (Fig. S2A). Thus, in agreement with previous data (19, 28), primary HIV-1 M Vpus inhibited NF-κB more efficiently than the NL4-3 or group N Vpu proteins. It has been reported that Vpu may suppress NF-κB activation by at least two different mechanisms: antagonism of tetherin (27) and stabilization of IκB and prevention of nuclear translocation of p65 (19). Thus, we also analyzed whether the TMD mutations affect the ability of Vpu to inhibit tetherin-mediated NF-κB stimulation. Consistent with previous data (27), tetherin expression induced NF-κB activation in a dose-dependent manner (Fig. S2B). However, wt and Tmut Vpu proteins suppressed tetherin-mediated NF-κB activation with similar potencies (Fig. S2B). This result is in agreement with our previous finding that primate lentiviral Vpu proteins efficiently prevent NF-κB activation independent of their antitetherin activity (19).

**FIG 2:**
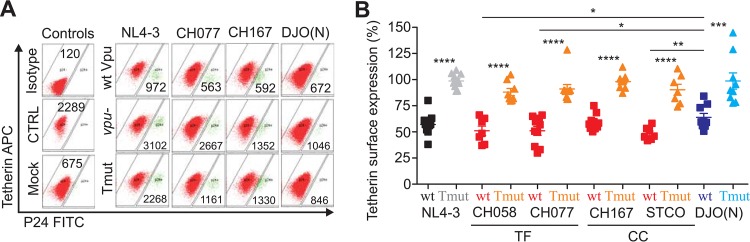
TMD mutations in Vpu disrupt tetherin down-modulation in HIV-1-infected primary T cells. PHA-activated PBMCs were infected with HIV-1 constructs containing wt, TMD mutated, or grossly defective *vpu* alleles and examined for tetherin surface expression 3 days later. (A) Examples of primary data. The numbers in the graphs give the mean fluorescence intensity (MFI) of tetherin expression in the HIV-1-infected (p24-positive) cell population. CTRL, control. (B) Levels of tetherin surface expression in cells infected with the wt and Vpu mutant constructs relative to those infected with the *vpu*-defective HIV-1 constructs (100%). Each symbol represents the result obtained for one individual PBMC donor investigated. Values that are significantly different are indicated by asterisks as follows: *, *P* < 0.05; **, *P* < 0.01; ***, *P* < 0.001; ****, *P* < 0.0001.

To determine the effects of the TMD mutations on the ability of Vpu to reduce tetherin and CD4 surface expression levels in primary human cells, phytohemagglutinin (PHA)-stimulated peripheral blood mononuclear cells (PBMCs) were infected with the six sets of HIV-1 infectious molecular clones containing wt, TMD mutated, or defective *vpu* genes. The latter contained either a 120-bp deletion (NL4-3) or two premature stop codons at positions 2 and 3 of the reading frame (all other IMCs). Three days later, the cells were stained for surface tetherin and CD4, permeabilized, and stained for intracellular p24 expression. On average, wt group M Vpus reduced the surface levels of tetherin by ~50%, while HIV-1 N Vpu achieved a reduction of 34% (Fig. 2). Since no specific antibodies are available, we could not determine whether the modest activity of the N-Vpu was the result of poor activity or low expression levels. The two Ala-to-Leu substitutions in the TMD domain of Vpu generally disrupted tetherin down-modulation in HIV-1-infected primary cells (Fig. 2). In contrast, all 18 HIV-1 IMCs efficiently reduced CD4 cell surface expression, irrespective of the *vpu* allele (see Fig. S3 in the supplemental material). This is because these proviral HIV-1 constructs express functional Env and Nef proteins and the latter protein particularly is highly effective in down-modulating CD4 in HIV-1-infected T cells (29).

### Tetherin antagonism is critical for effective HIV-1 production in CD4^+^ T cells.

CD4^+^ T cells are the first productively infected cell type detected in primary HIV-1 infection (30) and TF HIV-1 strains, which establish *de novo* clinical infection, are less sensitive to inhibition by type I interferon (IFN) than chronic controls are (31, 32). To determine the role of Vpu-mediated tetherin antagonism in virus production and sensitivity to IFN, we infected activated CD4^+^ T cells with equivalent amounts of virus in the presence and absence of IFN-α and determined the levels of p24 antigen production in culture supernatants on day 7 postinfection. Because of their importance in HIV-1 transmission, we focused on the CH058-TF and CH077-TF viruses and used the NL4-3 and chronic CH167-CC IMCs as controls.

**FIG 3:**
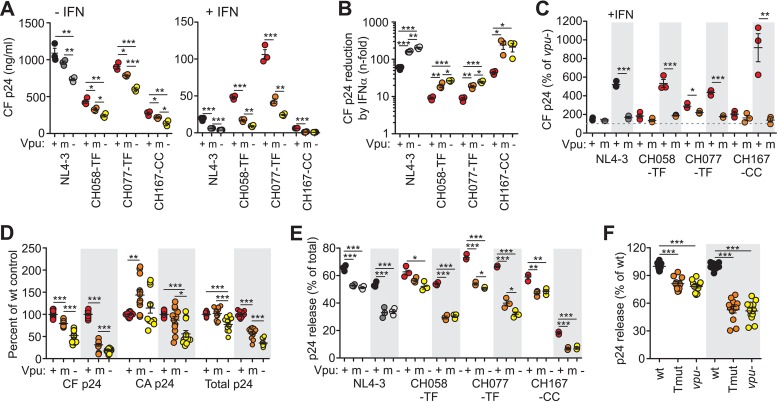
Effects of alterations in *vpu* on HIV-1 yield and release in CD4^+^ T cells in the presence or absence of IFN-α. (A) Cell-free (CF) p24 antigen levels in the supernatant of CD4^+^ T cells at day 7 postinfection with HIV-1 IMCs expressing wt (+), Tmut (m), or no (−) Vpu proteins. Virus yield was determined after triplicate HIV-1 infection in the presence of 500 U/ml IFN-α (+) and absence of IFN-α (−). (B) Reduction of cell-free p24 antigen yield by IFN-α treatment. For calculation of *n*-fold reduction, the levels of p24 antigen obtained in the absence of IFN were divided by those obtained in the presence of IFN-α. (C) Enhancement of p24 release by wt and Tmut Vpu proteins in the presence (shaded) or absence of exogenous IFN-α. Data were derived from the experiment shown in panel A. The levels of cell-free p24 antigen relative to the cultures infected with the respective *vpu*-defective HIV-1 IMCs (100%, indicated by the dashed line) are shown. (D) Cell-free, cell-associated (CA), and total p24 yield in CD4^+^ T cells infected with HIV-1 NL4-3, CH058-TF, CH077-TF, and CH167 IMCs containing wt, mutant, or grossly defective *vpu* genes. The average values obtained for the respective wt IMCs were set at 100%. (E) Efficiency of p24 release in CD4^+^ T cells infected with the indicated HIV-1 IMCs. Values present percentages of cell-free p24 antigen out of the total p24 detected in the presence (shaded) and absence of IFN-α. Cell-free and cell-associated p24 antigen were quantified by an enzyme-linked immunosorbent assay (ELISA) at day 7 postinfection. (F) Effect of TMD mutations in Vpu or entire lack of Vpu function on the efficiency of virion release. Values obtained for all four IMCs analyzed are shown relative to the respective wt viruses (100%). Values that are significantly different are indicated by asterisks as follows: *, *P* < 0.05; **, *P* < 0.01; ***, *P* < 0.001.

In agreement with published data (31, 32), the CH058-TF and CH077-TF HIV-1 IMCs produced substantially higher levels of cell-free virus than the chronic CH167-CC or the T-cell-line-adapted NL4-3 construct in the presence, but not in the absence, of IFN-α (Fig. 3A). IFN-α treatment reduced cell-free p24 yield of wt TF HIV-1 IMCs by ~9-fold. This reduction was significantly lower than that observed for NL4-3 (58.2-fold) and the CC CH167-CC IMC (44.1-fold) (Fig. 3B). Point mutations in the TM domain of Vpu increased IFN sensitivity to an extent similar (~3.1-fold) to that of the complete lack of Vpu (~3.5-fold) (Fig. 3B). In the absence of IFN-α, wt CH058-TF and CH077-TF Vpus enhanced p24 production by 85% and 189%, whereas the corresponding Tmut Vpus achieved only 38% and 121% (Fig. 3C). The ability of Vpu to enhance cell-free p24 levels was more pronounced in the presence of IFN-α: wt CH058-TF, CH077-TF, and NL4-3 Vpu proteins increased cell-free p24 antigen yield about 5-fold (Fig. 3C). In agreement with its potent antitetherin activity in transient-transfection assays (Fig. 1C), the CH167-CC Vpu achieved a 9-fold enhancement (Fig. 3C), although the corresponding IMC produced only low levels of cell-free virus (Fig. 3A). Tmut Vpus increased the levels of p24 antigen in the supernatants of IFN-α CD4^+^ T-cell cultures only marginally compared to HIV-1 IMCs lacking Vpu function entirely (Fig. 3C). Our finding that wt Vpu proteins enhanced the levels of cell-free HIV-1 TF viruses in the presence of IFN-α substantially more efficiently than Tmut Vpus is consistent with a relevant role of tetherin antagonism for viral spread *in vivo*.

### IFN-α treatment impairs release of *vpu* mutant but not wt HIV-1 strains.

To assess the effects of the TMD mutations on total virus production and the efficiency of virion release, we determined the levels of cell-associated and total p24 antigen in the HIV-1-infected cultures (see Fig. S4A and S4B in the supplemental material). Total p24 was determined as the sum of both cell-free and cell-associated p24. The impact of Vpu on the levels of cell-associated p24 varied (Fig. S4C), most likely because functional *vpu* genes may also enhance viral replication and thus increase the total number of infected cells. Fully functional wt Vpus increased the total amount of p24 antigen produced in IFN-treated cultures by ~3-fold, and this enhancement was severely impaired by the TMD mutations in Vpu (Fig. 3D). We quantified released p24 as the ratio of cell-free p24 divided by total p24. IFN-α treatment generally decreased the efficiency of virus release (Fig. 3E). TMD mutations or the lack of Vpu function reduced virion release efficiency by ~20% in the absence of IFN-α treatment and by ~50% in the presence of IFN-α treatment (Fig. 3F). Although the Tmut Vpus failed to enhance virion release (Fig. 3E and F), they significantly enhanced total (Fig. 3D and Fig. S4B) p24 production in the infected cultures.

### TF IMCs produce high titers of cell-free virus even in the absence of Vpu function.

**FIG 4:**
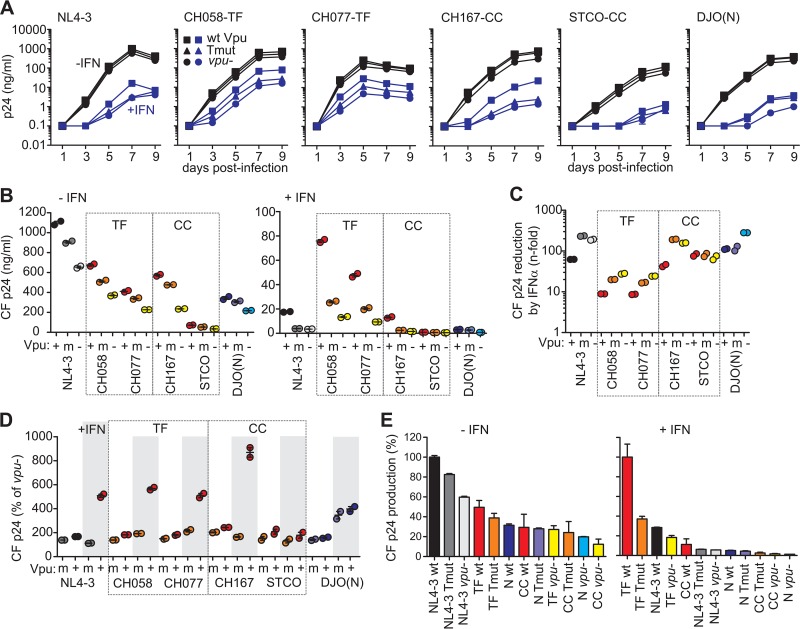
Replication of wt and *vpu* mutant HIV-1 constructs in CD4^+^ T cells in the presence (+) and absence (−) of IFN-α. (A) Replication kinetics of HIV-1 IMCs expressing wt, TMD mutant, or no Vpu proteins in CD4^+^ T cells in the presence of 500 U/ml IFN-α (blue lines) or absence of IFN-α (black lines). Results show median values of p24 antigen production (*n* = 3) from two different donors. (B) Cumulative p24 antigen levels in the presence and absence of IFN-α measured at 1, 3, 5, 7, and 9 days postinfection. Panels B, C, and D show the results obtained from two different blood donors. (C) Reduction of cumulative cell-free p24 antigen yield by IFN-α treatment. (D) Enhancement of cumulative p24 yield by wt and Tmut Vpu proteins in the presence (shaded) or absence of exogenous IFN-α. Data were derived from the experiment shown in panel A. Values present total cell-free virus yield relative to the respective *vpu*-defective HIV-1 IMC (100%). (E) Ranking of wt and *vpu* mutant or defective HIV-1 IMCs according to their efficiency in cell-free p24 production. The levels achieved for the most potent IMC were set at 100%. Values are median values of p24 antigen production (plus SEM [error bars]; *n* = 3).

The data outlined above suggest that in addition to the antitetherin function, other activities of M-Vpus contribute to efficient viral replication in primary CD4^+^ T cells. However, the results shown in Fig. 3 were derived from only a single time point (day 7) following HIV-1 infection. To further examine the importance of Vpu-mediated tetherin antagonism for HIV-1 replication, we monitored virus production in primary CD4^+^ T cells infected with wt and *vpu* mutant HIV-1 IMCs over a period of 9 days (Fig. 4A). In addition, we included another CC HIV-1 IMC (STCO-CC) and the HIV-1-N DJO0131 clone in the analyses. As expected, TF HIV-1 strains CH058-TF and CH077-TF exhibited substantially higher levels of virus production than the remaining IMCs in the presence of IFN-α (Fig. 4A and B). On average, IFN-α treatment decreased virus yield of these two TF viruses ~9-fold, whereas IFN-α treatment decreased the yields of the CC HIV-1 strains CH167-CC and STCO-CC 47- and 75-fold, and IFN-α treatment of the group N virus resulted in a reduction of more than 100-fold (Fig. 4C).

The TMD mutations in Vpu resulted in cell-free virus yields that were intermediate between wt and *vpu*-defective HIV-1 group M strains in the absence of IFN-α treatment (Fig. 4B). In the presence of IFN-α, the Tmut Vpus failed to enhance the p24 levels in cultures infected with NL4-3 or the CC strains and had only modest effects on the two TF strains (Fig. 4B). Mutations in the TMD or lack of Vpu function enhanced sensitivity of most HIV-1 M IMCs to IFN-α inhibition ~3- to 4-fold (Fig. 4C). The exception was the STCO-CC strain, which showed low levels of replication and was highly susceptible to IFN inhibition irrespective of Vpu function (Fig. 4C). The single group N virus was also very susceptible to IFN inhibition. Notably, the ~4-fold enhancement of p24 production by HIV-1 N Vpu in the presence of IFN was not impaired by the TMD mutations (Fig. 4A to C). Thus, N-Vpu appears to promote HIV-1 replication independent of its modest antitetherin activity. In contrast, the 5- to 9-fold enhancing effect of group M Vpus was disrupted by the TMD mutations (Fig. 4D). We ranked the HIV-1 IMCs based on their efficacy to produce cell-free virus (Fig. 4E). In the absence of IFN, the T-cell-line-adapted NL4-3 construct showed the highest virus yield and functional *vpu* genes had only modest effects on the levels of cell-free p24 (Fig. 4E). In contrast, TF HIV-1 IMCs produced the highest levels of cell-free p24 in the presence of IFN. Mutations in the TMD domain or entire loss of Vpu function reduced cell-free p24 yield from TF IMCs by 2.5- and 5-fold, respectively. However, even the *vpu* mutated or *vpu*-defective TF IMCs showed higher virus yields than the CC HIV-1 group M and group N strains (Fig. 4E, right). Thus, Vpu-mediated tetherin antagonism is critical for high virus yield from infected CD4^+^ T cells in the presence of IFN-α, but additional *vpu*-independent functions also play a role.

### TF IMC infected cells release virions with high efficacy even in the absence of Vpu function.

**FIG 5:**
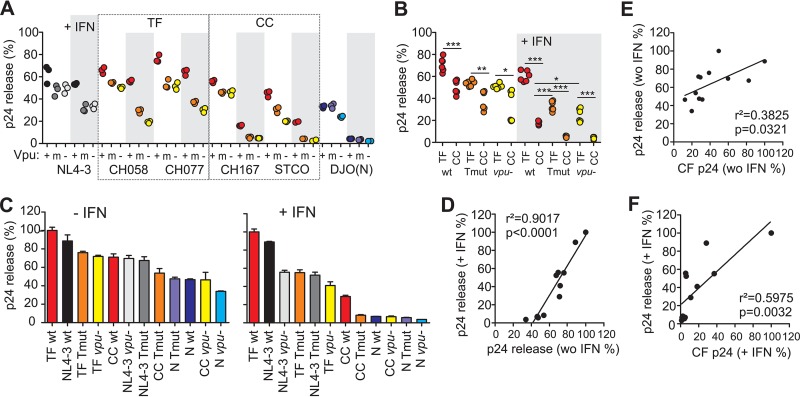
Release of wt and *vpu* mutant HIV-1 constructs in CD4^+^ T cells in the presence and absence of IFN-α. (A) Values present percentages of cell-free p24 antigen out of the total p24 detected in the presence (shaded) and absence of IFN-α. Results from triplicate infections of T cells derived from three PBMC donors are shown. Cell-free and cell-associated p24 antigen was quantified by ELISA at day 5 postinfection. (B) Efficiency of TF and CC virus release in CD4^+^ T cells infected with the indicated HIV-1 IMCs. Values present percentages of cell-free p24 antigen out of the total p24 detected in the presence (shaded) and absence of IFN-α. (C) Ranking of wt and *vpu* mutant or defective HIV-1 IMCs according to their release efficiency. The levels achieved by the most potent IMCs were set at 100%. Values are median values of release efficacy (plus SEM [error bars]; *n* = 3). (D) Correlation between the virus release efficiencies measured in the presence and absence (without [wo]) of IFN-α. (E and F) Correlation between the virus release efficiencies (values derived from panel C) and p24 antigen yield (values derived from Fig. 4E) in the absence (E) and presence (F) of IFN-α.

Next, we determined the levels of cell-free and total p24 antigen in the cultures (see Fig. S5 in the supplemental material) to calculate the efficiency of virus release. Unexpectedly, the Tmut HIV-1 M IMCs produced total quantities of p24 antigen that were as high (CH058-TF and STCO-CC) or slightly higher (NL4-3; CH077-TF and CH167-CC) than the p24 antigen amounts produced by the respective wt viruses (Fig. S5B), which may be due to more-effective cell-to-cell spread and/or Vpu-mediated degradation of CD4 in Tmut infected cultures. In agreement with data shown in Fig. 3, IFN-α treatment reduced the efficiency of virus release, particularly in the absence of a functional Vpu. Furthermore, release of the TF HIV-1 IMCs was more efficient than that of CC viruses, while release of the HIV-1 group N DJO0131 IMC was markedly reduced relative to the five group M viruses (Fig. 5A). These differences in virion release were highly reproducible in independent experiments (Fig. S6). Interestingly, Tmut as well as *vpu*-defective TF HIV-1 strains showed significantly higher efficiencies of virion release than the wt CC HIV-1 strains in the presence of IFN-α (Fig. 5A and B). In general, the differences in virion release capacity were much more pronounced in IFN-α-treated T-cell cultures than in untreated CD4^+^ T-cell cultures (Fig. 5C), but the relative efficiencies of the 18 HIV-1 IMCs measured under both conditions showed a highly significant correlation (Fig. 5D). CC HIV-1 IMCs containing disrupted or mutated *vpu* genes and all HIV-1 group N constructs exhibited very low (<10% of wt TF HIV-1 IMCs) efficiencies of virion release (Fig. 5C, right). For all viruses, there was a significant correlation between p24 production and release, particularly in IFN-α-treated cultures (Fig. 5E and F), although other factors clearly also influence virus production. Finally, we examined whether the human-specific adaptations in Vpu affected the infectiousness of viral particles produced in the infected CD4^+^ T-cell cultures. We found that the TF-derived virions were substantially more infectious than the CC- and group N-derived particles (Fig. S7A). The mutations in Vpu, however, had no significant effect on virion infectivity (Fig. S7B). Altogether, these results suggest that high infectivity and efficient virion release might represent hallmarks of TF HIV-1 strains and that the latter is only partly dependent on potent Vpu-mediated tetherin antagonism.

## DISCUSSION

Great apes transmitted SIVs to humans on at least four independent occasions. However, only one of these transmission events resulted in a pandemically spreading pathogen (1). Elucidating the viral properties that mediate efficient spread of HIV-1 is important for preventive strategies. It has been suggested that the acquisition of Vpu-mediated tetherin antagonism promoted efficient spread of HIV/AIDS (10, 11). However, direct evidence for this hypothesis has been lacking because thus far only T-cell-line-adapted viruses have been characterized that were completely Vpu deficient. Here, we show that amino acid mutations in the TMD domain of Vpu, which are critical for antitetherin activity, reduce virion production and release in the presence of IFN-α by about 50%. Tmut TF viruses were released about 3-fold more efficiently than Tmut CC viruses, and >10-fold more efficiently than the Tmut group N virus in IFN-α-treated human CD4^+^ T cells, although this release was only partly dependent on Vpu (Fig. 5C). Thus, our data support the hypothesis that adaptation at key Vpu residues that confer effective tetherin antagonism were indeed important for the spread of HIV/AIDS. Moreover, our data suggest that TF HIV-1 M strains have evolved additional yet-to-be-defined Vpu-independent functions to ensure efficient virus release and replication in the face of an innate antiviral response.

The TMD mutations in Vpu resulted in HIV-1 M virus levels that were intermediate between wt and *vpu*-defective IMCs, although in the presence of exogenous IFN-α, this phenotype was almost identical to that of HIV-1 lacking Vpu entirely (Fig. 4A and 5B). However, in the absence of IFN-α, Tmut Vpus had little, if any, reducing effect on the total levels of HIV-1 p24 antigen production (see Fig. S3B and Fig. S4B in the supplemental material). The remaining activity of Tmut Vpus is unlikely due to residual antitetherin activity, since there were no significant differences in the amounts of particle release from TMD-mutated and Vpu-deficient IMC infected cultures (Fig. 3F and 5A). Together, these data suggest that both the newly acquired antitetherin activity and other Vpu functions that are conserved between HIV-1 and SIVcpz Vpus, such as degradation of CD4 or inhibition of NF-κB activation, increase viral replication fitness in primary CD4^+^ T cells. Lentiviral accessory proteins are well-known for their multifunctionality, only some of which might be lost after cross-species transmission.

Although mutations in the TMD domain of Vpu and complete lack of Vpu function reduced the replication potential and particle release of TF viruses, particularly upon treatment with IFN-α, their growth rates and virion production capacity remained significantly higher than those of wt CC HIV-1 strains (Fig. 4 and 5). Tetherin-independent effects on virus release are further supported by the reduced replication capacity of the CH167-CC IMC compared to the two TF viruses and HIV-1 NL4-3 (Fig. 3A and 4A), although its Vpu showed the highest potency in antagonizing tetherin (Fig. 1C) and enhancing p24 production (Fig. 3C and 4D). Moreover, the Vpu proteins of TF viruses are equally potent at antagonizing human tetherin as those derived from CC HIV-1 strains (33, 34). Thus, other as-yet-unknown viral properties that promote efficient release of virions from infected T cells likely contribute to virus spread. One of these properties is the ability to potently degrade and down-modulate CD4. It has been shown that CD4 inhibits virus release (35) and reduces virion infectivity (36, 37). The phenotype of the HIV-1 group N DJO0131 strain that lacks a Vpu-mediated CD4 degradation function (12) supports these findings. Potent CD4 down-modulation is also consistent with previous data showing that TF virions are slightly more infectious and contain about twofold-more Env per particle than CC viruses do (32). Although all IMCs efficiently down-modulated cell surface CD4 due to functional Nef and Env expression, Vpu-mediated CD4 degradation may contribute to potent virus release and replication by preventing intracellular interaction between CD4 and the viral Env glycoprotein (35–37). However, other cellular factors that affect virion release efficacy, such as T-cell immunoglobulin (Ig) and mucin domain (TIM) proteins (38), may also play a role, and it will be interesting to determine whether they are efficiently counteracted by TF HIV-1.

It is still unclear whether cell-free or cell-associated virus predominates in sexual HIV-1 transmission (39, 40), although multiple studies found a correlation between the efficiency of transmission and the level of cell-free virus in blood or genital secretions (41–43). We found that TF viruses produced much higher levels of cell-free virus than CC HIV-1 M, whereas the levels of cell-associated virus were higher in the T-cell cultures infected with the CH167-CC and group N DJO0131 viruses (see Fig. S5A in the supplemental material). Thus, it is possible that cell-free HIV-1 plays an important role in sexual virus transmission.

The group N HIV-1 molecular clone was highly sensitive to IFN inhibition and produced very little cell-free virus in the presence of IFN (Fig. 4E), although the levels of cell-associated p24 antigen and total produced virus were comparable to those of the two TF HIV-1 M strains both in the presence and absence of IFN-α (see Fig. S5 in the supplemental material). However, in the presence of IFN, both wt and TMD mutated group N Vpus increased cell-free virus production about fourfold (Fig. 4D). Thus, it seems clear that the DJO (N) Vpu promotes virus production by yet-to-be-defined tetherin-independent mechanisms. Whether these effects of N-Vpu contribute to viral pathogenesis remain to be determined, but it is noteworthy that HIV-1 N strains can cause CD4^+^ T-cell depletion and AIDS (44–46).

In summary, our results demonstrate that Vpu-mediated tetherin antagonism enhances virus production and release from primary CD4^+^ T cells by about fivefold in the presence of high levels of type I IFN. We further show that even *vpu*-defective or mutated TF HIV-1 strains exhibit higher virion release capacity than wt CC HIV-1 strains in IFN-α-treated primary T cells. Thus, TF HIV-1 M *vpu* genes appear to encode functions in addition to effective tetherin antagonism that enhance viral replication and release in the presence of IFN. Finally, CD4 T cells infected with wt group N virus produced about 4-fold-less cell-free virions compared to CC HIV-1 M strains and about 13-fold-less virions compared to TF HIV-1 M IMCs. Thus, the efficiency with which virus is released from infected CD4 T cells appears to be correlated with the ability of HIV-1 to spread in humans, with antitetherin activity playing a major role at least for group M viruses.

## MATERIALS AND METHODS

### HIV-1 proviral constructs.

Generation of NL4-3, CH058, CH077, CH167, STCO, and group N DJO0131 HIV-1 IMCs has been previously described (12, 25, 26, 32, 47) (Table 1). Site-directed mutagenesis of *vpu* was performed by splice overlap extension PCR, and all constructs were verified by sequence analysis. TMD mutations in Vpu are shown in Fig. 1A. Grossly *vpu*-defective IMCs contained a premature stop codon at amino acid positions 2 and 3 of the *vpu* reading frame, except NL4-3 that contained a 120-bp deletion in *vpu*.

### Expression vectors.

Cloning of HIV-1 *vpu* genes and human *tetherin*, *CD4*, *NTB-A*, and *CD1d* alleles into cytomegalovirus (CMV) promoter-based expression vectors coexpressing the green fluorescent protein (GFP) or not was performed as described previously (5, 12).

### Cell culture.

HEK293T cells were maintained in Dulbecco’s modified Eagle medium (DMEM) supplemented with 10% heat-inactivated fetal bovine serum, 350 µg/ml l-glutamine, 100 µg/ml streptomycin sulfate, and 100 U/ml penicillin. HEK293T cells were transfected by the calcium phosphate method. Human peripheral blood mononuclear cells (PBMCs) from healthy donors were isolated using lymphocyte separation medium (Biocoll separating solution; Biochrom), stimulated for 3 days with PHA (1 µg/ml), and cultured in RPMI 1640 medium with 10% fetal calf serum (FCS) and 10 ng/ml interleukin 2 (IL-2) prior to infection.

### Flow cytometric analysis.

To determine the effects of Vpu on CD4, CD1d, NTB-A, and tetherin cell surface expression, HEK293T cells were transfected by the calcium phosphate method with 1 µg of a CD4, CD1d, NTB-A, or tetherin expression vector and 5 µg of pCG eGFP/Vpu constructs expressing eGFP alone or together with Vpu. Two days posttransfection, CD4, CD1d, NTB-A, or tetherin expression was examined by fluorescence-activated cell sorting (FACS) analysis. An allophycocyanin (APC)-conjugated anti-human tetherin antibody (BioLegend), APC-conjugated anti-human CD4 antibody (catalog no. MHCD0405; Invitrogen), a phycoerythrin-conjugated anti-CD1d antibody (catalog no. 550255; BD Biosciences), or an APC-conjugated anti-SLAM6 antibody (catalog no. FAB19081A; R&D Systems) was used for staining. Fluorescence of stained cells was detected by two-color flow cytometry and Vpu-mediated CD4, CD1d, NTB-A, or tetherin down-modulation was calculated as described previously for the functional analysis of *nef* alleles (48). To determine the effect of Vpu on tetherin surface expression levels in primary cells, PHA-stimulated PBMCs were transduced by spinoculation (2 h at 37°C, 1,300 × *g*) with vesicular stomatitis virus glycoprotein G (VSVg)-pseudotyped HIV-1 proviral constructs. Three days after transduction, PBMCs were dual stained for surface tetherin (allophycocyanin-conjugated anti-human tetherin antibody from BioLegend) and CD4 (phycoerythrin-conjugated anti-human CD4 [catalog no. MHCD0404; Invitrogen]), permeabilized. and stained intracellularly for p24 with a fluorescein isothiocyanate (FITC)-conjugated antibody (Beckman Coulter).

### Western blot.

To monitor Vpu expression, HEK293T cells were transfected with 5 µg of vector DNA coexpressing enhanced GFP (eGFP)- and AU-1-tagged Vpus. The *vpu* alleles were not codon optimized. Two days posttransfection, cells were harvested, lysed in coimmunoprecipitation (CO-IP) buffer (150 mM NaCl, 50 mM HEPES, 5 mM EDTA, 0.1% NP-40, 0.5 mM sodium orthovanadate, 0.5 mM NaF [pH 7.5]), and cell lysates were separated in 4 to 12% bis-Tris gels (Invitrogen). After gel electrophoresis, proteins were transferred onto polyvinylidene difluoride (PVDF) membranes and probed with AU-1 antibody (catalog no. MMS-130P; Covance). Subsequently, blots were probed with anti-mouse or anti-rabbit IRDye Odyssey antibodies (catalog no. 926-32210 and 926-32221; LI-COR), and proteins were detected using a LI-COR Odyssey scanner. For internal controls, blots were incubated with antibodies specific for eGFP (catalog no. 290-50; Abcam) and β-actin (catalog no. 8227-50; Abcam).

### Tetherin antagonism in HEK293T cells.

To determine the capability of Vpu to antagonize tetherin, HEK293T cells were seeded in six-well plates and transfected with 2 µg of NL4-3 Δ*vpu* internal ribosomal entry site (IRES) eGFP, 500 ng of Vpu expression plasmid, and different dilutions of tetherin expression plasmid. A pCG vector expressing eGFP only was used to equalize the DNA concentrations. At 2 days posttransfection, supernatants were harvested, and the yield of infectious HIV-1 was determined by a 96-well infection assay on TZM-bl indicator cells as described previously (49).

### Inhibition of NF-κB activity.

To determine the effect of Vpu on NF-κB activity, HEK293T cells in 96-well format were cotransfected in triplicates with 0.1 µg of firefly luciferase reporter construct under the control of three NF-κB binding sites, 0.025 µg of *Gaussia* luciferase construct under the control of a minimal pTAL promoter for normalization, and 0.04 µg of expression vectors for a mutant of IKKβ containing two phosphomimetic changes (S177E and S181E) in the activation loop that render the expressed protein constitutively active or increasing the concentration of tetherin, as well as 0.025 µg pCG eGFP/Vpu. Dual-luciferase assays were performed 48 h posttransfection, and the firefly luciferase signals were normalized to the internal *Gaussia* luciferase control as previously described (19).

### Viral replication in CD4^+^ T cells.

To assess the contribution of tetherin antagonism to the IFN resistance of full-length infectious molecular clones (IMCs), we generated virus stocks of wild-type, TMD mutant (Tmut), and *vpu*-defective IMCs by transfection of 293T cells. CD4^+^ T cells were positively selected (Miltenyi Biotec) from buffy coats from blood samples from three healthy donors (Research Blood Components, LLC). Cells were activated by anti-CD2/CD3/CD28 beads (Miltenyi Biotec) and cultured in cell culture medium (RPMI 1640 medium containing 15% fetal bovine serum [FBS], 1× penicillin−streptomycin−l-glutamine [PSG] plus IL-2 [30 U/ml]) for 4 days at 37°C and 5% CO_2_. Cells were pooled and either treated with 500 U/ml of IFN-α2 (PBL Assay Science) or left untreated. Cells were infected with normalized amounts of virus in small volumes (250 µl) overnight (12 to 15 h). Cells were washed with phosphate-buffered saline (PBS) (three times) and resuspended in cell culture medium. Every 48 h, supernatants were sampled for cell-free p24 measurements, and medium (containing IFN or not containing IFN) was added back. To quantify cell-associated p24, we harvested cells at days 7 and 9 and resuspended cells in lysis buffer. Cell-free and cell-associated p24 antigen levels were quantified using the commercially available p24 AlphaLisa detection kit (PerkinElmer). Each virus was tested in duplicate per experiment, and experiments were repeated twice in two separate pools of CD4^+^ T cells.

### Virion infectivity.

A total of 8,300 TZM-bl cells were seeded per well in a 96-well plate. At a confluence of ~40%, the cells were infected with 100 µl of cell-free supernatant of infected CD4^+^ T cells obtained 7 days postinfection in the presence of DEAE-dextran (final concentration, 40 µg/ml). Forty-eight hours later, the cells were lysed with Cell Culture Lysis Reagent (catalog no. E153A; Promega), lysates were frozen at −80°C for 2 h, and relative light units (RLU) were determined using the luciferase assay system (Promega). The RLUs obtained were normalized to the capsid antigen p24 levels to obtain RLUs per picogram of p24 capsid antigen. Each measurement was performed in duplicate.

### Ethics statement.

Ethical approval for the utilization of human-derived cells was obtained from the Ethics Committee of Ulm University Medical Center.

### Statistical analysis.

Statistical calculations were performed using two-tailed unpaired Student’s *t* tests (for comparison of different groups) or paired Student’s *t* tests using GraphPad Prism version 5.0.

## SUPPLEMENTAL MATERIAL

Figure S1  Expression and tetherin, CD4, NTB-A, and CD1a down-modulation activities of TMD mutant Vpu proteins. (A) HEK293T cells were transfected with plasmids encoding the indicated AU-1-tagged Vpus and analyzed by Western blotting. An empty vector and mock-transfected cells were used as negative controls. The *vpu* alleles were not codon optimized. (B) FACS analysis of HEK293T cells cotransfected with tetherin or CD4 expression vectors and pCG plasmids expressing eGFP alone (lanes 2 and 3) or together with the indicated *vpu* allele. The mean fluorescence intensities (MFIs) are indicated. (C to E) Vpu-dependent reduction of CD4 (C), NTB-A (D), and CD1d (E) and surface expression in HEK293T cells. Shown are the levels of receptor cell surface expression relative to those measured in cells transfected with the eGFP control vector. Values are mean values (±SEM) derived from three experiments. Wild-type *vpu* alleles are indicated by dark colors, and Tmut Vpu proteins are indicated by light colors. Download Figure S1, PDF file, 0.1 MB

Figure S2  Inhibition of NF-κB activation by wt and Tmut Vpu proteins. (A) HEK293T cells were cotransfected with the indicated *vpu* alleles, a firefly luciferase reporter construct under the control of three NF-κB binding sites, a *Gaussia* luciferase construct for normalization, and expression vectors for a constitutively active mutant of IKKβ as inducer of NF-κB. Luciferase activities were determined 48 h posttransfection. Values are mean values (±SEM) derived from three experiments. (B) HEK293T cells were transfected as described above for panel A, except that different quantities of tetherin expression vectors were used to induce NF-κB activation. Download Figure S2, PDF file, 0.02 MB

Figure S3  Down-modulation of CD4 in PBMCs infected with HIV-1 IMCs differing in their *vpu* coding sequences. PHA-activated PBMCs were transduced with the indicated VSVg-pseudotyped HIV-1 IMCs and examined for CD4 surface expression 3 days later. (A) Examples of primary FACS data. Numbers give mean fluorescence intensities (MFI) of CD4 expression in the HIV-1-infected (p24+) cell population. (B) Levels of surface expression in virally infected (p24+) cells relative to uninfected cells (100%). Each symbol provides the result obtained for one individual PBMC donor. Download Figure S3, PDF file, 0.1 MB

Figure S4  Effects of alterations in *vpu* on cell-associated and total HIV-1 yield in the presence and absence of IFN-α. (A and B) Cell-associated (A) and total (B) p24 antigen levels in CD4^+^ T cells at day 7 postinfection with HIV-1 IMCs expressing wt (+), Tmut (m), or no (−) Vpu proteins. p24 levels were determined by ELISA after triplicate HIV-1 infection in the presence of 500 U/ml IFN-α (right) and absence of IFN-α (left). (C and D) Enhancement of cell-associated (C) and total (D) p24 antigen levels by wt and Tmut Vpu proteins in the presence (shaded) or absence of exogenous IFN-α. Data were derived from the experiment shown in panels A and B. The levels of cell-associated and total p24 antigen relative to the cultures infected with the respective *vpu*-defective HIV-1 IMCs (100%, indicated by the dashed line) are shown. Download Figure S4, PDF file, 0.02 MB

Figure S5  Effects of alterations in *vpu* on cumulative cell-associated and total p24 production in the presence and absence of IFN-α. (A and B) Cumulative cell-associated (A) and total (B) p24 antigen levels in CD4^+^ T cells at 5, 7, and 9 days postinfection with HIV-1 IMCs expressing wt (+), Tmut (m), or no (−) Vpu proteins. p24 levels were determined by ELISA in the presence of 500 U/ml IFN-α (right) or absence of IFN-α (left). Download Figure S5, PDF file, 0.02 MB

Figure S6  Differences in virion release efficacy are highly reproducible. Correlation between the release efficiencies at day 7 postinfection in the experiment shown in Fig. 3E and average values obtained at 5, 7, and 9 days postinfection in an independent experiment (Fig. 5A) in the absence (left) and presence (right) of IFN-α treatment. Download Figure S6, PDF file, 0.02 MB

Figure S7  Infectivity of HIV-1 IMCs produced in infected CD4^+^ T cells. (A) Infectivity of HIV-1 IMCs expressing wt, Tmut, or no (−) Vpu proteins obtained from infected CD4^+^ T cells at day 7 postinfection. Values represent averages of duplicate infection and were obtained in the absence of IFN-α treatment. (B) Infectivity of the HIV-1 IMCs shown in panel A grouped based on their *vpu* coding sequences. The minimum and maximum values, 25% and 75% percentiles, and median values are shown. Download Figure S7, PDF file, 0.02 MB
