# Supplementary figures and images for: Vpu-Mediated Counteraction of Tetherin Is a Major Determinant of HIV-1 Interferon Resistance
Source: mBio. 2016 Aug 16;7(4):e00934-16. doi: 10.1128/mBio.00934-16 (PMC4992969; doi:10.1128/mBio.00934-16)

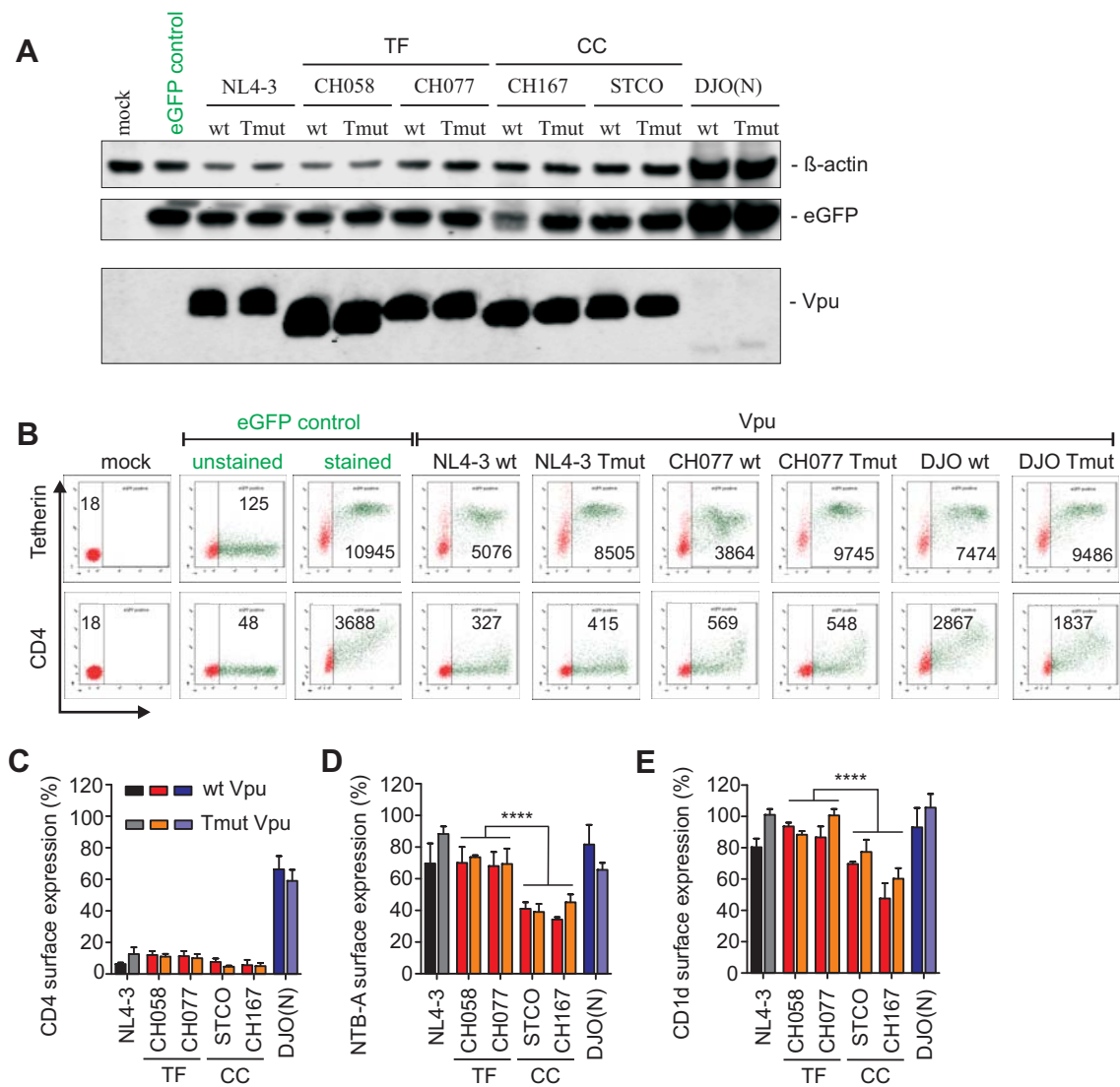

Supplement: Figure S1 — Expression and tetherin, CD4, NTB-A, and CD1a down-modulation activities of TMD mutant Vpu proteins. (A) HEK293T cells were transfected with plasmids encoding the indicated AU-1-tagged Vpus and analyzed by Western blotting. An empty vector and mock-transfected cells were used as negative controls. The vpu alleles were not codon optimized. (B) FACS analysis of HEK293T cells cotransfected with tetherin or CD4 expression vectors and pCG plasmids expressing eGFP alone (lanes 2 and 3) or together with the indicated vpu allele. The mean fluorescence intensities (MFIs) are indicated. (C to E) Vpu-dependent reduction of CD4 (C), NTB-A (D), and CD1d (E) and surface expression in HEK293T cells. Shown are the levels of receptor cell surface expression relative to those measured in cells transfected with the eGFP control vector. Values are mean values (±SEM) derived from three experiments. Wild-type vpu alleles are indicated by dark colors, and Tmut Vpu proteins are indicated by light colors. Download [file mbo004162899sf1.pdf]

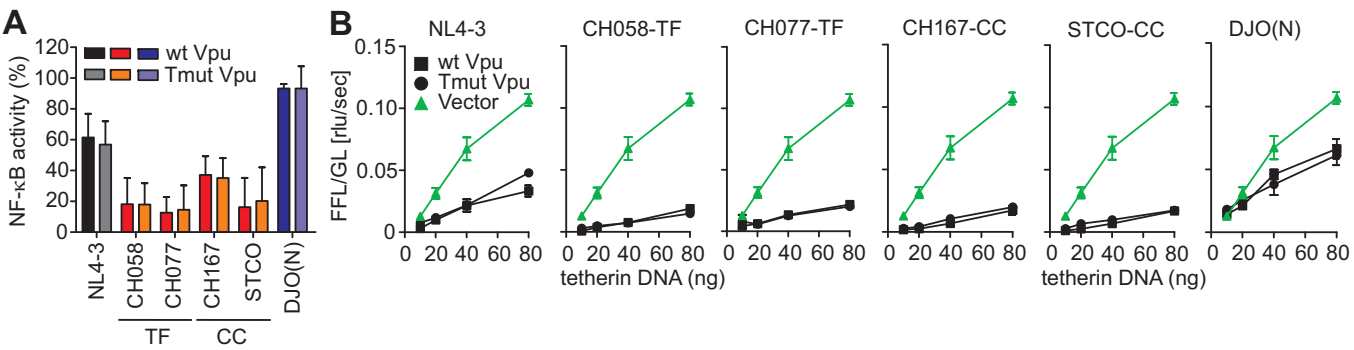

Supplement: Figure S2 — Inhibition of NF-κB activation by wt and Tmut Vpu proteins. (A) HEK293T cells were cotransfected with the indicated vpu alleles, a firefly luciferase reporter construct under the control of three NF-κB binding sites, a Gaussia luciferase construct for normalization, and expression vectors for a constitutively active mutant of IKKβ as inducer of NF-κB. Luciferase activities were determined 48 h posttransfection. Values are mean values (±SEM) derived from three experiments. (B) HEK293T cells were transfected as described above for panel A, except that different quantities of tetherin expression vectors were used to induce NF-κB activation. Download [file mbo004162899sf2.pdf]

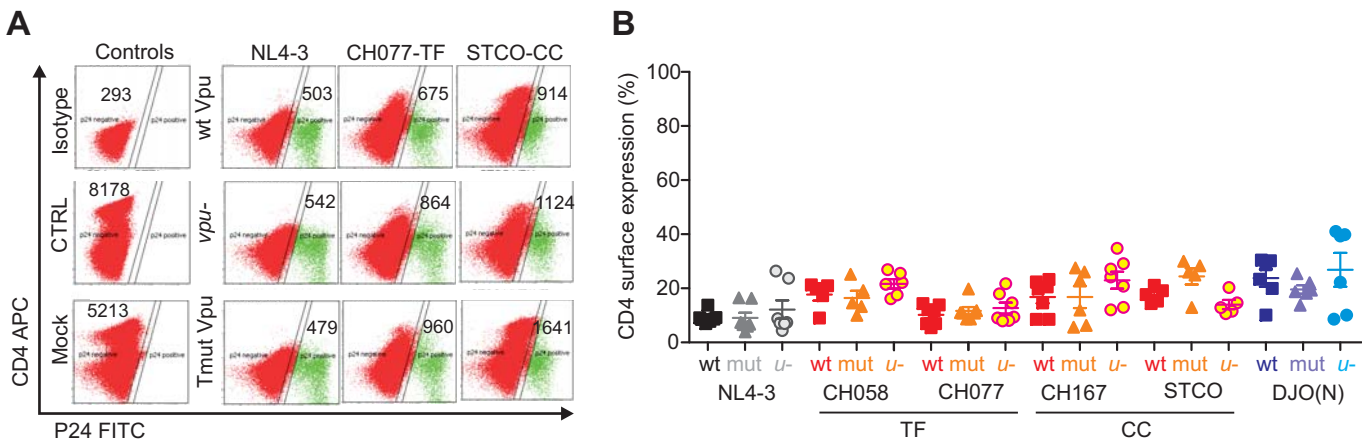

Supplement: Figure S3 — Down-modulation of CD4 in PBMCs infected with HIV-1 IMCs differing in their vpu coding sequences. PHA-activated PBMCs were transduced with the indicated VSVg-pseudotyped HIV-1 IMCs and examined for CD4 surface expression 3 days later. (A) Examples of primary FACS data. Numbers give mean fluorescence intensities (MFI) of CD4 expression in the HIV-1-infected (p24+) cell population. (B) Levels of surface expression in virally infected (p24+) cells relative to uninfected cells (100%). Each symbol provides the result obtained for one individual PBMC donor. Download [file mbo004162899sf3.pdf]

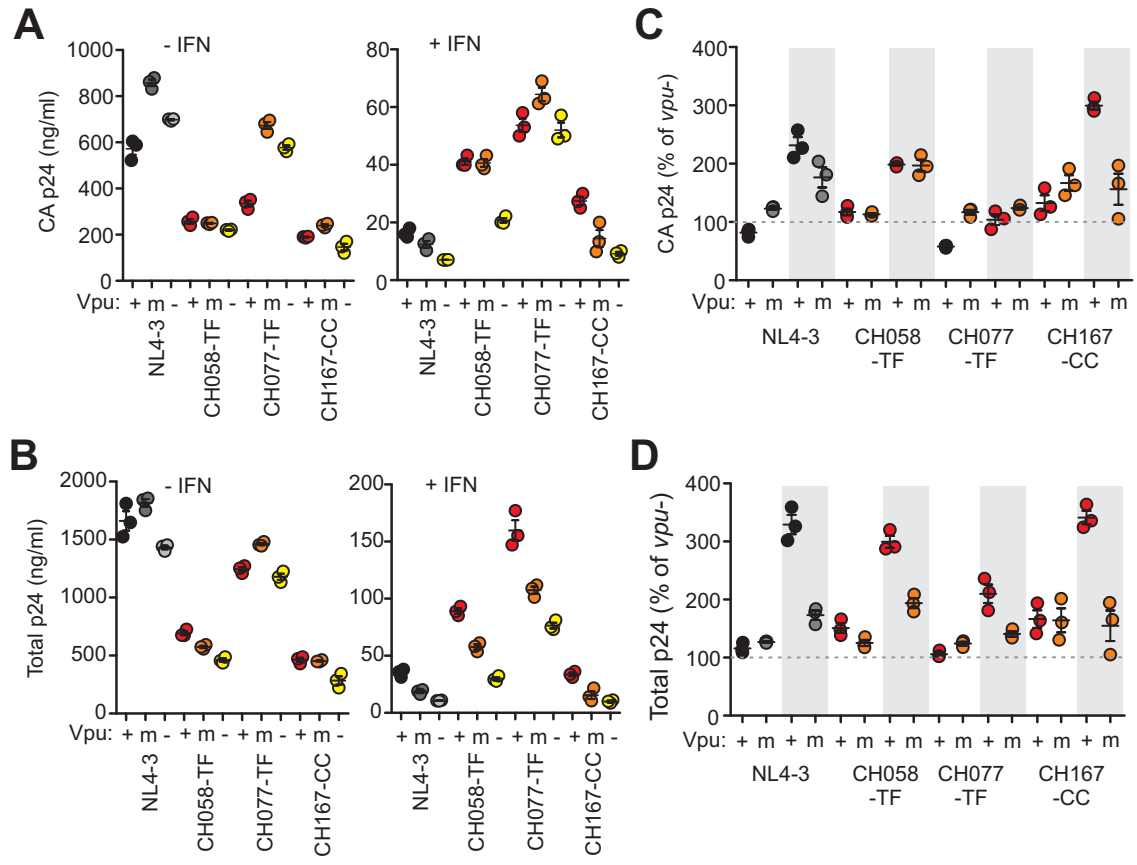

Supplement: Figure S4 — Effects of alterations in vpu on cell-associated and total HIV-1 yield in the presence and absence of IFN-α. (A and B) Cell-associated (A) and total (B) p24 antigen levels in CD4+ T cells at day 7 postinfection with HIV-1 IMCs expressing wt (+), Tmut (m), or no (−) Vpu proteins. p24 levels were determined by ELISA after triplicate HIV-1 infection in the presence of 500 U/ml IFN-α (right) and absence of IFN-α (left). (C and D) Enhancement of cell-associated (C) and total (D) p24 antigen levels by wt and Tmut Vpu proteins in the presence (shaded) or absence of exogenous IFN-α. Data were derived from the experiment shown in panels A and B. The levels of cell-associated and total p24 antigen relative to the cultures infected with the respective vpu-defective HIV-1 IMCs (100%, indicated by the dashed line) are shown. Download [file mbo004162899sf4.pdf]

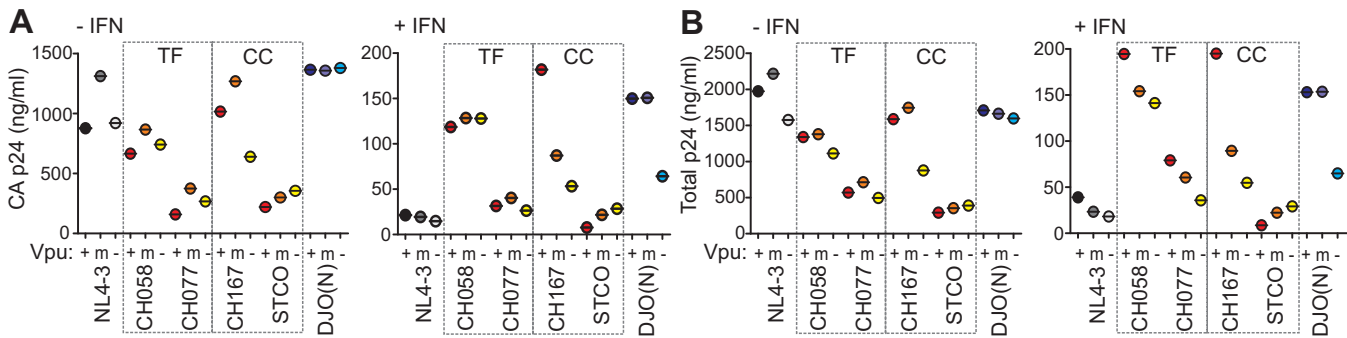

Supplement: Figure S5 — Effects of alterations in vpu on cumulative cell-associated and total p24 production in the presence and absence of IFN-α. (A and B) Cumulative cell-associated (A) and total (B) p24 antigen levels in CD4+ T cells at 5, 7, and 9 days postinfection with HIV-1 IMCs expressing wt (+), Tmut (m), or no (−) Vpu proteins. p24 levels were determined by ELISA in the presence of 500 U/ml IFN-α (right) or absence of IFN-α (left). Download [file mbo004162899sf5.pdf]

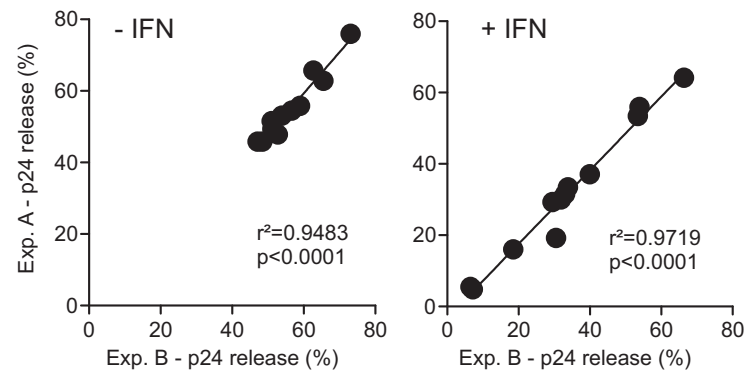

Supplement: Figure S6 — Differences in virion release efficacy are highly reproducible. Correlation between the release efficiencies at day 7 postinfection in the experiment shown in Fig. 3E and average values obtained at 5, 7, and 9 days postinfection in an independent experiment (Fig. 5A) in the absence (left) and presence (right) of IFN-α treatment. Download [file mbo004162899sf6.pdf]

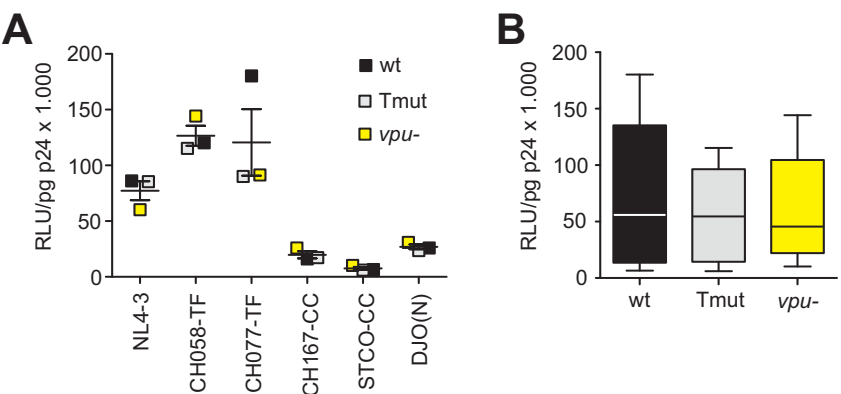

Supplement: Figure S7 — Infectivity of HIV-1 IMCs produced in infected CD4+ T cells. (A) Infectivity of HIV-1 IMCs expressing wt, Tmut, or no (−) Vpu proteins obtained from infected CD4+ T cells at day 7 postinfection. Values represent averages of duplicate infection and were obtained in the absence of IFN-α treatment. (B) Infectivity of the HIV-1 IMCs shown in panel A grouped based on their vpu coding sequences. The minimum and maximum values, 25% and 75% percentiles, and median values are shown. Download [file mbo004162899sf7.pdf]
